# Dengue

**Published:** 1894-12

**Authors:** Ira W. Porter

**Affiliations:** Sanford, Fla.


### DENGUE.

BY IRA W. PORTER, M. D., SANFORD, FLA.

The late epidemic of dengue fever in Florida has demonstrated nothing new as regards its predisposing or exciting cause. As far as I have learned, no abortive or prophylactic treatment has been successful. The condition of the weather seems to have had no effect on it. As yet we have had no frost, but the two storms that visited us at the autumnal equinox have had no effect on the disease. It occurs during the cool weather in the same degree as in the hot weather.

It has been a pandemic disease, extending from Key West to Jacksonville. Race and nationality seem to have little influence upon the disease. The white, the Cuban and the negro seem to suffer alike. Infants in arms and old people have alike succumbed to its rapacity; all classes of society alike have suffered. The physician has enjoyed no immunity. It has prevailed as a rule in the cities. There have been some cases in the country, but these are exceptions.

The period of incubation of the cases has, as a rule, been brief. The patient has suffered for a day or two with slight headache, lassitude, loss of appetite, furred tongue and slight muscular soreness, generally described as a tired feeling in the lower limbs as after a long walk.

All of the characteristic symptoms have not occurred in all of the cases. Some times the peculiar rash would be absent; at other times the swollen glands in the neck would not appear, while the rash and a sore throat, with sore and stiff muscles, and an elevated temperature, would be the prominent symptoms. The bowels have been constipated in the majority of cases with irritable stomach, and, in some instances, bilious vomiting.

I have not seen the temperature above 103.2° F. in the mouth. The pulse has been full, hard and strong, ranging from 110 to 130.

The first paroxysm of fever abates suddenly with the occurrence of profuse sweats or copious evacuations of the bowels; these latter being dark, greenish, and foul-smelling I have seen no epistaxis as is mentioned by Dr. Jas. C. Wilson, in his *Continued Fevers.*

This intermission has been of short duration, lasting only a few hours, and sometimes not more than two hours. The second paroxysm of fever is of short duration and not always accompanied by a rash. Muscular soreness remains, of course. The fever in this stage is not so high as in the first. The fever gradually subsides and leaves the patient in an enfeebled and exhausted condition.

I have had better success with this disease with the simplest forms of treatment. In the first paroxysm of fever, I give ten grains of phenacetine every two hours until the fever cools, which it does in a very few hours; antikamnia for the headache; and in the afternoon, the following pill:

℞. Hydrag. Chlor. Mitae, gr. iii.
Ext. Colocynth Comp., gr. iss.
Podophyllin, gr. ¼.
Ext. Hyoscyamus, gr. iss.
M. Fiat massa et div. pil. no. iii.
Sig.—One pill every two hours till all are taken.

This gives a copious and easy bilious action. If the muscular pains are severe, I give five grains of soda salicylate with the phenacetine. This usually cuts short the first paroxysm.

During the intermission, I give quinia and soda salicylate together. This does not prevent a return of fever, but in the cases in which I have tried it, it undoubtedly cut short the second paroxysm of fever and has the advantage of relieving the muscular pains to a great extent.

As soon as the fever begins to rise a second time, I begin on the phenacetine and soda salicylate again. This lowers the temperature in a few hours. If there is any muscular soreness remaining, I continue the soda salicylate for a day or two.

As a tonic after the fever, I give the following:

℞. Quinia Sulph., oz. iv.
Ferrum Redactum, gr. xxv.
Strych. Sulph, gr. ss.
Arsenious Acid, gr. ss.
M. Fiat massa et div. pil. no. xxv.
Sig.—One pill three times a day after meals.

I have been very successful with this treatment. In no case that I have had, has the attack lasted longer than four days at the outside, and I havehad no relapses.

I have not heard of any death that could be attributed to this disease. I know that the disease has not proven fatal in the section of country immediately surrounding Sanford.

---

Matrimony as a Cure.—A dress-reform advocate asserts that a man should not marry a lady whose waist measures less than twenty-five inches.—Indian Medical Record.

If the man is all he should be the waist will develop.—*Ex.*

---

An Excellent combination for cold in the head is said to consist of a snuff containing 2 grains of cocaine hydrochloride, 4 grains of menthol, 30 grains of boric acid and 8 grains of finely-powdered coffee. This is to be taken in pinches five or six times a day.—*Ex.*

